# Extensive astrocyte synchronization advances neuronal coupling in slow wave activity *in vivo*

**DOI:** 10.1038/s41598-017-06073-7

**Published:** 2017-07-20

**Authors:** Zsolt Szabó, László Héja, Gergely Szalay, Orsolya Kékesi, András Füredi, Kornélia Szebényi, Árpád Dobolyi, Tamás I. Orbán, Orsolya Kolacsek, Tamás Tompa, Zsombor Miskolczy, László Biczók, Balázs Rózsa, Balázs Sarkadi, Julianna Kardos

**Affiliations:** 1 Institute of Organic Chemistry, Research Centre for Natural Sciences, Hungarian Academy of Sciences, Magyar tudósok körútja 2, 1117 Budapest, Hungary; 2 Institute of Experimental Medicine, Hungarian Academy of Sciences, Szigony 43, 1083 Budapest, Hungary; 3 Institute of Enzymology, Research Centre for Natural Sciences, Hungarian Academy of Sciences, Magyar tudósok körútja 2, 1117 Budapest, Hungary; 4 MTA-ELTE Laboratory of Molecular and Systems Neurobiology, Department of Physiology and Neurobiology, Eötvös Loránd University, Pázmány Péter sétány 1C, 1117 Budapest, Hungary; 5 Institute of Materials and Environmental Chemistry, Research Centre for Natural Sciences, Hungarian Academy of Sciences, Magyar tudósok körútja 2, 1117 Budapest, Hungary; 6 Institute of Cancer Research, Medical University Wien, Borschkegasse 8a, 1090 Wien, Austria

## Abstract

Slow wave activity (SWA) is a characteristic brain oscillation in sleep and quiet wakefulness. Although the cell types contributing to SWA genesis are not yet identified, the principal role of neurons in the emergence of this essential cognitive mechanism has not been questioned. To address the possibility of astrocytic involvement in SWA, we used a transgenic rat line expressing a calcium sensitive fluorescent protein in both astrocytes and interneurons and simultaneously imaged astrocytic and neuronal activity *in vivo*. Here we demonstrate, for the first time, that the astrocyte network display synchronized recurrent activity *in vivo* coupled to UP states measured by field recording and neuronal calcium imaging. Furthermore, we present evidence that extensive synchronization of the astrocytic network precedes the spatial build-up of neuronal synchronization. The earlier extensive recruitment of astrocytes in the synchronized activity is reinforced by the observation that neurons surrounded by active astrocytes are more likely to join SWA, suggesting causality. Further supporting this notion, we demonstrate that blockade of astrocytic gap junctional communication or inhibition of astrocytic Ca^2+^ transients reduces the ratio of both astrocytes and neurons involved in SWA. These *in vivo* findings conclusively suggest a causal role of the astrocytic syncytium in SWA generation.

## Introduction

Increasing body of evidence substantiating the impact of astrocytes on neuronal activity prompted a paradigm shift from the neurocentric philosophy of nervous system function. Accordingly, astrocytes are increasingly recognized as major players in the modulation of neuronal function under both physiological^1–3^ and pathophysiological conditions^4–7^. Beyond the local astroglial control over synaptic activity^8–12^, however, little is known about the role of astrocytic networks in modulating large-scale neuronal ensembles. Exploration of the role of large-scale astrocytic networks in information processing and cognition still lags behind its neuronal counterpart^13, 14^. We conceived that fundamental properties of networking astrocytes may underlie physiological network-network interaction between astrocytes and neurons. Astrocytes are capable of 1) detecting neuronal activity, 2) responding to this activity by raising local Ca^2+^ transients, 3) propagating the local changes over extended spatial scales by Ca^2+^ waves traveling through the directly and densely interconnected astrocytic syncytium and 4) modulating neuronal activity at multiple locations by releasing gliotransmitters and other neuromodulatory substances or regulating ionic homeostasis^15^. Thus, astrocytes are ideally positioned to induce or contribute to synchronization of large-scale neuronal networks. Along this line, we have previously demonstrated that the astrocytic and neuronal networks are co-synchronized and the astrocytic network activation dynamics did significantly contribute to the emergent neuronal synchronization during seizure-like events^16^. We have also shown that disruption of long-range astrocytic coupling by gap junction blockade prevented the appearance of seizure-like events or significantly reduced their magnitude^16^. In the present study, we explored whether astrocytic networks may also play a role *in vivo* in the generation of a physiological synchronous neuronal activity, the slow-wave oscillations.

Slow-wave sleep is the deepest phase of non-rapid eye movement sleep, characterized by slow/delta wave oscillations at 0.5–2 Hz^17, 18^. The neuronal network-level oscillations observed by EEG or local field potential (LFP) measurements correspond to the fluctuations of neuronal membrane potentials between more silent, hyperpolarized DOWN states and more active, depolarized UP states, during which neuronal firing probability is highly increased. Although it is widely recognized that the slow wave activity (SWA) plays a crucial role in many important physiological functions, especially memory consolidation^19^, it is largely unknown what mechanisms are responsible for the emerging neuronal synchronization and its propagation from the cortex to various areas. Despite the early observations back in the 1960s showing that the active cell types during sleep are non-pyramidal^20^, even the identity of the cells specifically activated in SWA is still mainly unclear.

Here we explored whether astrocytic and neuronal networks are co-activated during SWA by using a recently developed transgenic rat line stably expressing the Ca^2+^-sensitive fluorescent protein GCaMP2^21^ in astrocytes and interneurons. We have observed that not only neurons, but also astrocytes show concurrent network-level activity during UP states. Importantly, extensive (>70%) involvement of the astrocytic network appeared earlier than that of the neuronal network, suggesting causality. Moreover, blockade of gap junctions reduced the magnitude of slow waves and specific blockade of either astrocytic Cx43 connexins or astrocytic Ca^2+^ signalling decreased the number of both neurons and astrocytes activated during UP states. Conclusively, these observations strongly support the contribution of astrocytes to SWA generation and substantiate the role of astrocytic activation in coordinating synchronized network activity.

## Results

### GCaMP2 is expressed in astrocytes and interneurons

Physiological activation of astrocytes *in vivo* can be followed by genetically encoded Ca^2+^ sensors^22, 23^. To explore the potential activation and interference of networking astrocytes with the neuronal activity during slow-wave sleep, we used a novel stable transgenic rat line expressing the Ca^2+^-sensitive fluorescent protein GCaMP2 under the control of a non-specific, synthetic CAG promoter^21, 24^. The cell types expressing GCaMP2 were identified by immunostaining and electrophysiological characterization.

**Figure 1:**
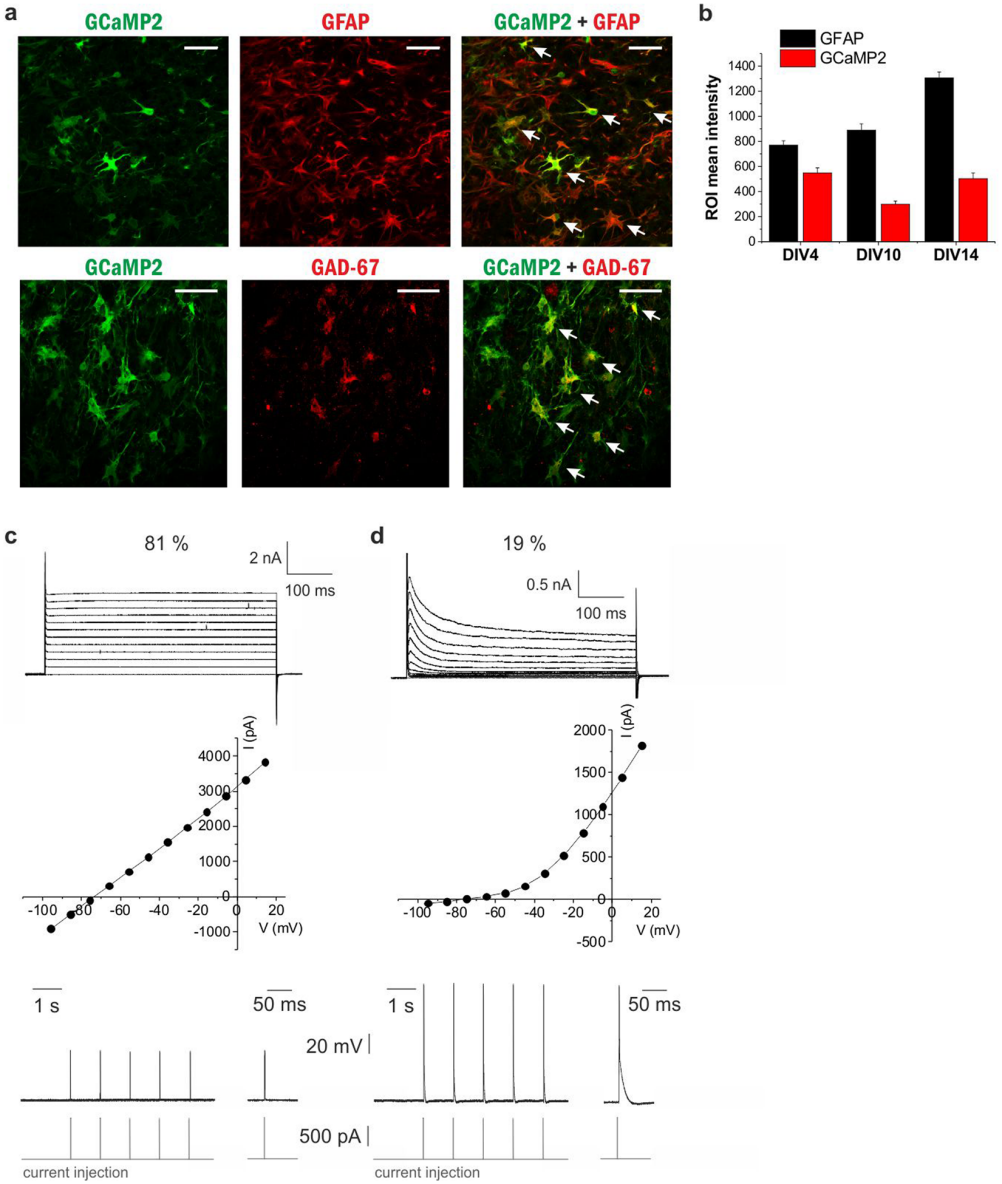
GCaMP2 is expressed in astrocytes and interneurons (**A**) Astrocytic and neuronal localization of GCaMP2 protein in organotypic cortical slices as shown by colocalization with the astrocyte-specific GFAP and interneuron-specific GAD-67 markers. Cells showing colocalization are marked by arrows. Scale bars: 50 μm. (**B**) Changes in GCaMP2 and GFAP expression as measured by average intensity within cell ROIs (n = 22–34 cells) in organotypic cortical slices as a function of days in culture (DIV). (**C**,**D**) Representative traces (top), I/V plot (middle) and action potential generating ability (bottom) of whole-cell patched GCaMP2 expressing astrocytes (**C**, n = 30) and neurons (**D**, n = 7) in organotypic cortical slices.

GCaMP2 immunoreactivity was found in all regions of organotypic cortical and hippocampal slices prepared from the transgenic rats (Fig. 1A, Supplementary Fig. 1). Importantly, many of the GCaMP2-positive cells were also stained by the astrocyte-specific marker anti-GFAP (Fig. 1A, Supplementary Fig. 1A), suggesting that GCaMP2 is expressed in astrocytes. After 10 days in culture (DIV10) 37.5% of total DAPI-labeled cells were expressing both GCaMP2 and GFAP in layer 2 (Pearson’s coefficient of colocalization was calculated to be 0.581). Since astrogliosis is known to occur in organotypic slices and GFAP overexpression is a hallmark of reactive astrocytes^25^, we explored whether GCaMP2 is expressed in reactive or non-reactive astrocytes. In line with increasing astrogliosis, we found that GFAP expression has been increasing during slice culturing (Fig. 1B). However, GCaMP2 expression level did not increase, instead slightly decreased over time (Fig. 1B), validating that GCaMP2 is expressed in non-reactive astrocytes. Moreover, we investigated whether GCaMP2 is expressed in microglia, another glial class that is activated in organotypic slice cultures. Anti-Iba1, a specific marker of microglia, however, did not colocalize with GCaMP2 (Supplementary Fig. 1A).

To the contrary, GCaMP2 immunostaining was found to be colocalized in both the cortex and the hippocampus with anti-GAD67, a specific marker of GABAergic interneurons (Fig. 1A, Supplementary Fig. 1A). DIV10) 16.7% of total DAPI-labeled cells were expressing both GCaMP2 and GAD67 (Pearson’s coefficient of colocalization was calculated to be 0.470). In addition, we also observed partial colocalization of the neuronal marker anti-NeuN with GCaMP2-positive cells, especially in the str. radiatum of the hippocampus (Supplementary Fig. 1A), where majority of the interneurons reside^26^. Importantly, however, both GCaMP2-immunoreactivity (Supplementary Fig. 1A), as well as native GCaMP2 expression was lacking in the str. pyramidale (Supplementary Fig. 1B), suggesting that GCaMP2 is not expressed in pyramidal cells.

To validate the immunohistochemical data on the expression of GCaMP2 in both neurons and astrocytes, we investigated electrophysiological characteristics of GCaMP2 expressing cells in organotypic cortical (n = 37 cells) and hippocampal (n = 65 cells) slices prepared from P6 rats by performing whole-cell recordings. Cells were patch clamped and the presence or absence of voltage-dependent currents was investigated in voltage clamp mode by depolarizing current pulses. The I-V curve of 81% of GCaMP2 expressing cells (n = 30) was found to be linear (Fig. 1B) with low input resistance (53.1 ± 8.0 MΩ), suggesting that they were astrocytes^27, 28^. Average membrane potential of astrocytes was −78.1 ± 1.9 mV in line with previous studies^27, 29, 30^ and injection of positive currents did not induce action potentials in these cells (Fig. 1C). The other subset of GCaMP2 expressing cells (n = 7) displayed markedly different I–V curve (Fig. 1C), with clear voltage-dependent currents and significantly higher input resistance (268.0 ± 69.6 MΩ). Furthermore, in contrast to cells characterized as astrocytes, these cells were able to generate action potentials in response to current injection in current clamp mode (Fig. 1C). Average membrane potential of these cells was found to be −76.4 ± 4.0 mV.

Data from organotypic hippocampal slices showed very similar results. 75% of cells (n = 49) were found to be astrocytes with average membrane potential of −73.4 ± 1.9 mV and average input resistance of 85.2 ± 15.3 MΩ. The other 25% of cells (n = 16) were classified as neurons with average membrane potential of −78.7 ± 3.6 mV and average input resistance of 307.7 ± 42.4 MΩ.

It is to note that the input resistance of astrocytes we measured here is slightly higher than classically reported in acute slices from adult animals^29, 31^. This divergence is reasonably attributed to the age of the animals (P6) used for preparing organotypic cultures. Expression of Kir4.1 channels has been shown to be increasing during development^30^, resulting in lower input resistance. Furthermore, relative distribution of low-resistance passive astrocytes and higher-resistance variably rectifying astrocytes is also drastically changing during the first 2–3 weeks of development in favor of lower resistance in adult animals^29, 32^. Notably, the low (<100 MΩ) astrocytic input resistance complements immunohistochemical characterization and further confirms that GCaMP2 is not expressed in reactive astroyctes, since their input resistance is substantially higher^33^.

In summary, *in vitro* data from immunohistochemical and electrophysiological studies confirmed that our transgenic rat line expresses GCaMP2 in both astrocytes and interneurons, two populations of neural cells that both may play a key role in generating synchronous brain activity.

### *In vitro* characterization of GCaMP2 response to pharmacological manipulation

**Figure 2:**
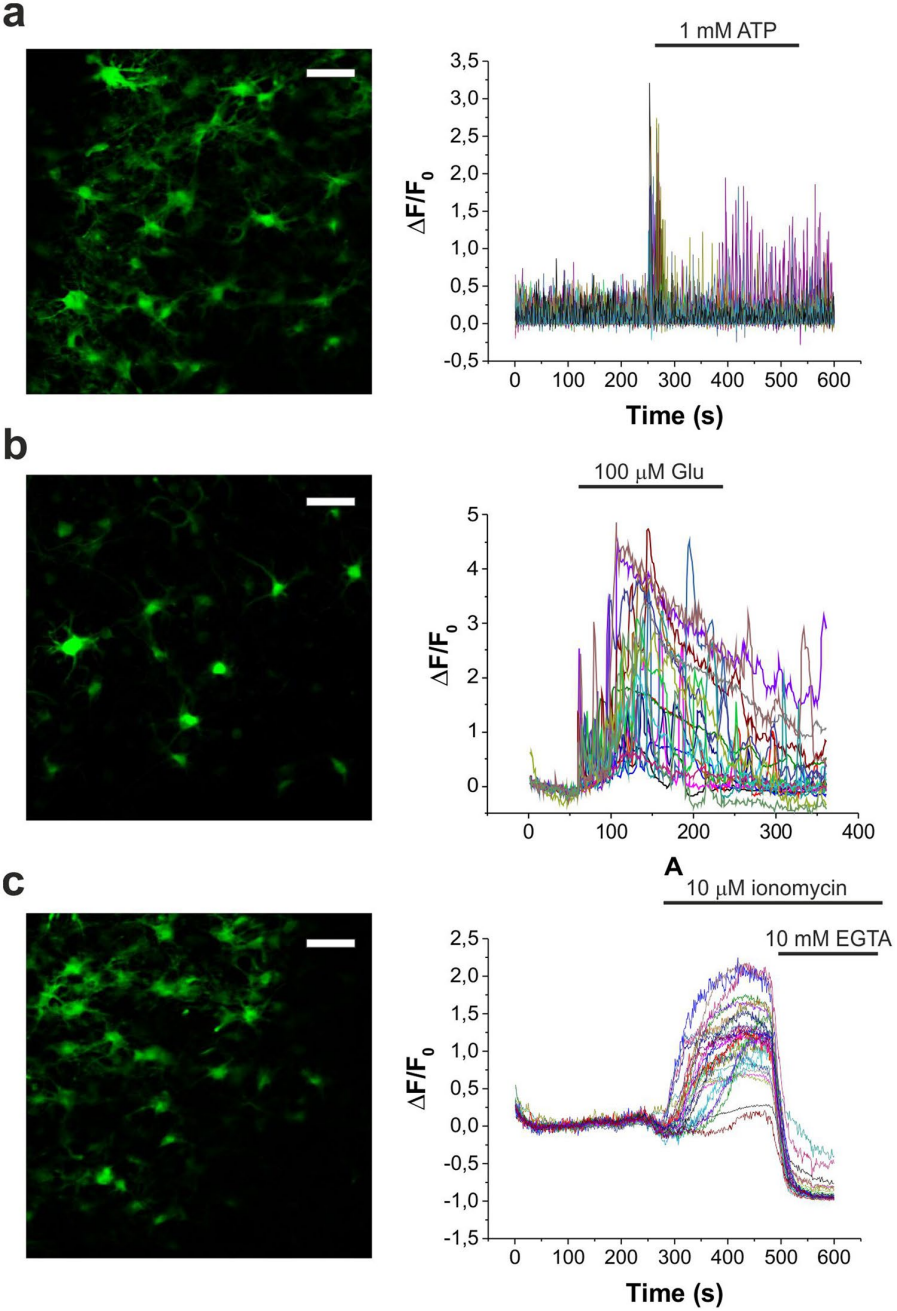
Pharmacological characterization of GCaMP2 response in organotypic cortical slices *in vitro*. (**A–C**) Representative fluorescent intensity changes of CAG-GCaMP2 in response to 1 mM ATP (**A**, n = 3), 100 μM Glu **(B**, n = 3**)** and 10 μM ionomycin without and with 10 mM Ca^2+^ chelating ethylene glycol tetraacetic acid (EGTA) **(C**, n = 3). Scale bars: 50 μm.

To evaluate the potential of GCaMP2 expressing transgenic rats to monitor changes in intracellular Ca^2+^ concentration, organotypic cortical and hippocampal slices were subjected to pharmacological tools inducing [Ca^2+^] increase in both neurons and astrocytes (Fig. 2, Supplementary Fig. 2, Supplementary Information Movie 1–3). Bath application of ATP triggered a significant increase in GCaMP2 fluorescence in all cells, which gradually declined thereafter (Fig. 2A). 100 µM glutamate (Glu) induced repetitive Ca^2+^ transients (Fig. 2B) in both soma and dendrites (see also Supplementary Information Movie 2). Finally, application of the Ca^2+^ ionophore ionomycin resulted in progressive increase in GCaMP2 fluorescence corresponding to accumulation of intracellular Ca^2+^. The fluorescence increase could be reversed by application of the Ca^2+^ chelator ethylene glycol tetraacetic acid (EGTA) (Fig. 2C).

Together, these results indicate that Ca^2+^ signalling is unimpeded in GCaMP2-expressing transgenic animals.

### Both astrocytes and neurons are activated during SWA *in vivo*

**Figure 3:**
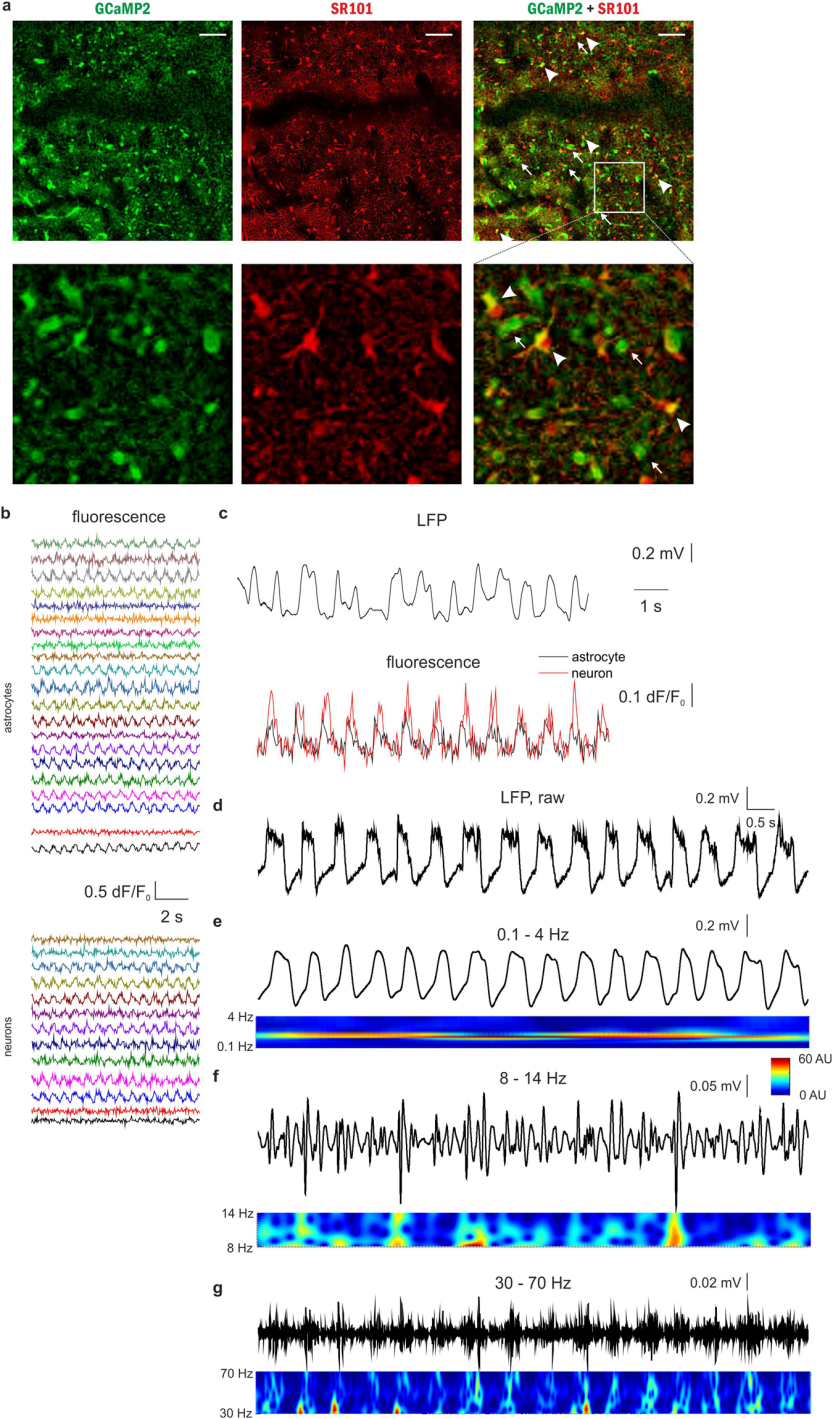
Both astrocytes and neurons are active during UP states *in vivo* in the rat primary visual cortex V1. (**A**) *Top:* Expression of GCaMP2 (green), labeling of astrocytes with SR101 (red). Both neurons (some marked by arrows) and astrocytes (some marked by arrowheads) express GCaMP2. *Bottom:* Zoomed-in image of the area marked by white rectangle at the top row. Scale bars: 100 µm. (**B**) 10-sec segments of ∆F/F_0_ fluorescent intensity traces of all identified astrocytes (n = 21) and neurons (n = 13) in the imaged area. (**C**) Simultaneous recording of local field potential (LFP, filtered at 0.1–4 Hz) and corresponding Ca^2+^ transients in a neuron (black) and an astrocyte (red) during SWA under ketamine*-*xylazine anesthesia. (**D–G**) Characteristic LFP activity in the slow wave (0.1–4 Hz), spindle (8–14 Hz) and gamma (30–70 Hz) frequency ranges as filtered LFP recordings (top) and their wavelet transformations (bottom). All data were recorded from the V1 area of a P76 rat (n = 15 imaging session, 60 s each).

In order to simultaneously measure the activity of neuronal and astroglial networks *in vivo*, rats expressing GCaMP2 were anesthetized with 100 mg/kg ketamine and 5 mg/kg xylazine, a well-known model of slow-wave sleep that reproduces major characteristics of SWA^34^. Discrimination of astrocytic and neuronal expression of GCaMP2 was done by administration of sulforhodamine-101 (SR101) either i.v. (10 mM, 100 μl) or onto the surface of the cerebral cortex during the cranial window preparation (200 μM, 2 μl). Cells showing both GCaMP2 fluorescence and SR101 labeling were identified as astrocytes, while cells displaying only GCaMP2 fluorescence were classified as neurons (Fig. 3A, Supplementary Fig. 3). Fluorescence intensity of neurons and astrocytes (3 to 65 cells per field of view of which 67 ± 8% were astrocytes) were continuously monitored in layer 2 of the V1 cortical area. Interestingly, we observed that during SWA, not only neurons, but also astrocytes displayed spontaneous recurrent fluorescent peaks corresponding to intracellular Ca^2+^ transients with approximately 1 Hz frequency (Fig. 3B,C). To explore whether these recurrent Ca^2+^ transients correspond to an increased activity during UP states of slow-wave sleep, we performed parallel field potential recordings in the same area. Both neuronal and astroglial Ca^2+^ transients coincided with positive half-waves of the LFP signal (Fig. 3B). Spectral analysis of the LFP signal showed that both spindle (8–14 Hz) and gamma (30–70 Hz) range activities were also time-locked to the positive half-waves (Fig. 3D–G). Since gamma range activity is known to be coupled to UP states^34^, these results confirm that the positive half-waves represent the UP states, therefore increased astrocytic activity appears in parallel with enhanced neuronal firing. Noteworthy, neither neuronal nor astroglial Ca^2+^ transients were observed when the animals were anesthetized with fentanyl (0.05 mg/kg, n = 5) or lower dose of ketamine/xylazine (50 and 5 mg/kg, respectively, n = 4).

In conclusion, the astrocytic network has been found to be participating in the SWA simultaneously with neuronal firing in UP states.

### Extensive synchronization emerges in the astrocytic network earlier than in the neuronal one

**Figure 4:**
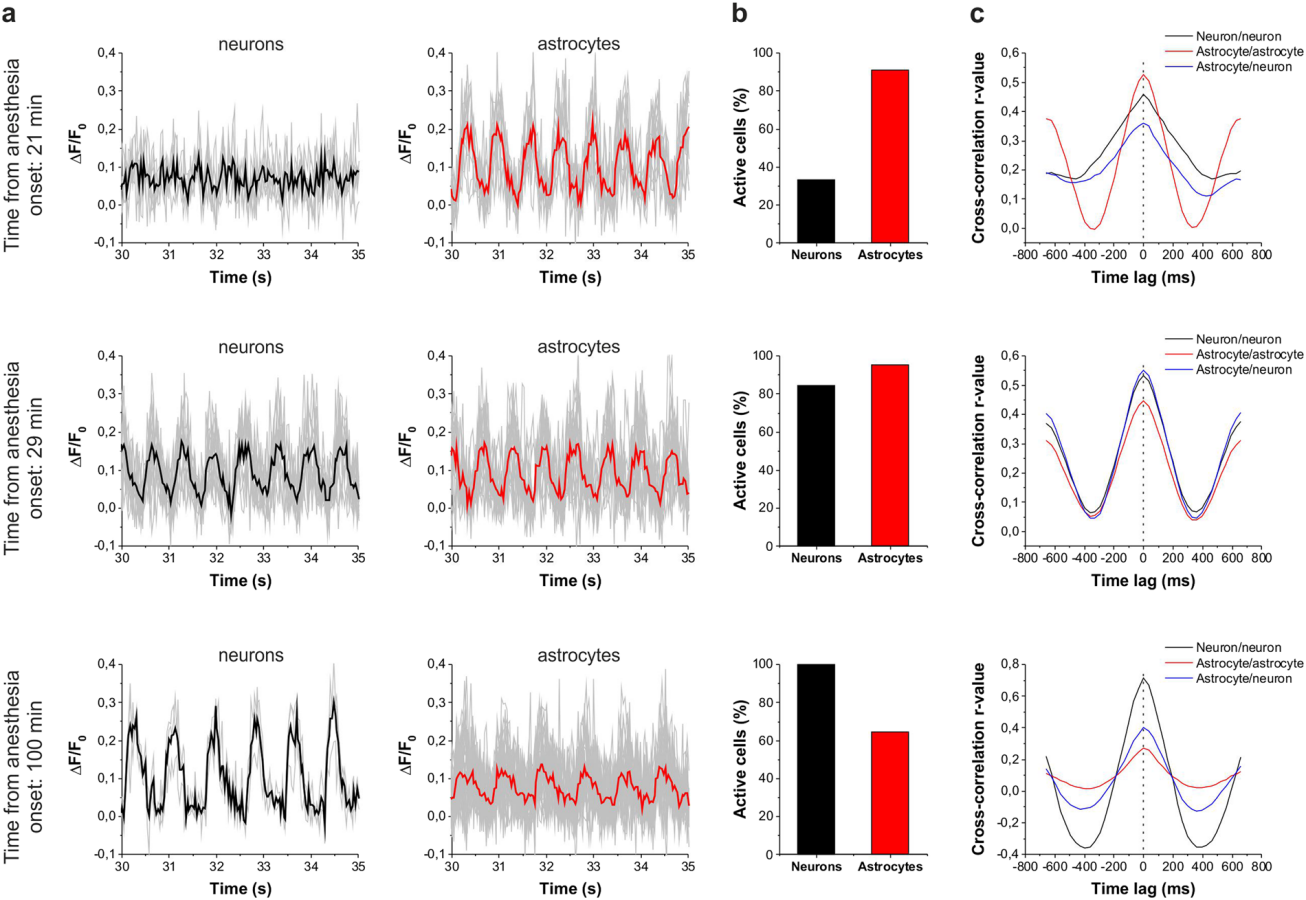
Astrocytes are more broadly involved in SWA than neurons in the early stage of ketamine/xylazine anesthesia. (**A–C**) Changes in the involvement of neurons and astrocytes in the network activity during SWA under ketamine*-*xylazine anesthesia after 21 min, 29 min and 100 min from the onset of anesthesia. (**A**) Fluorescent intensity changes detected in neurons and SR101-identified astrocytes. Individual Ca^2+^ traces (gray) and their averages in neurons (black) and astrocytes (red) are shown on 5-sec segments of the imaging sessions at each time point. (**B**) The ratio of neurons (black) and astrocytes (red) showing repetitive Ca^2+^ transients in percentage of all astrocytes or neurons, respectively, in the field of view in this particular imaging session. (**C**) Cross-correlation of neuron/neuron (black), astrocyte/astrocyte (red) and astrocytes/neuron (blue) pairs of active cells (n = 12–420 cell pairs). All data were recorded from the V1 area of a P78 rat.

**Figure 5:**
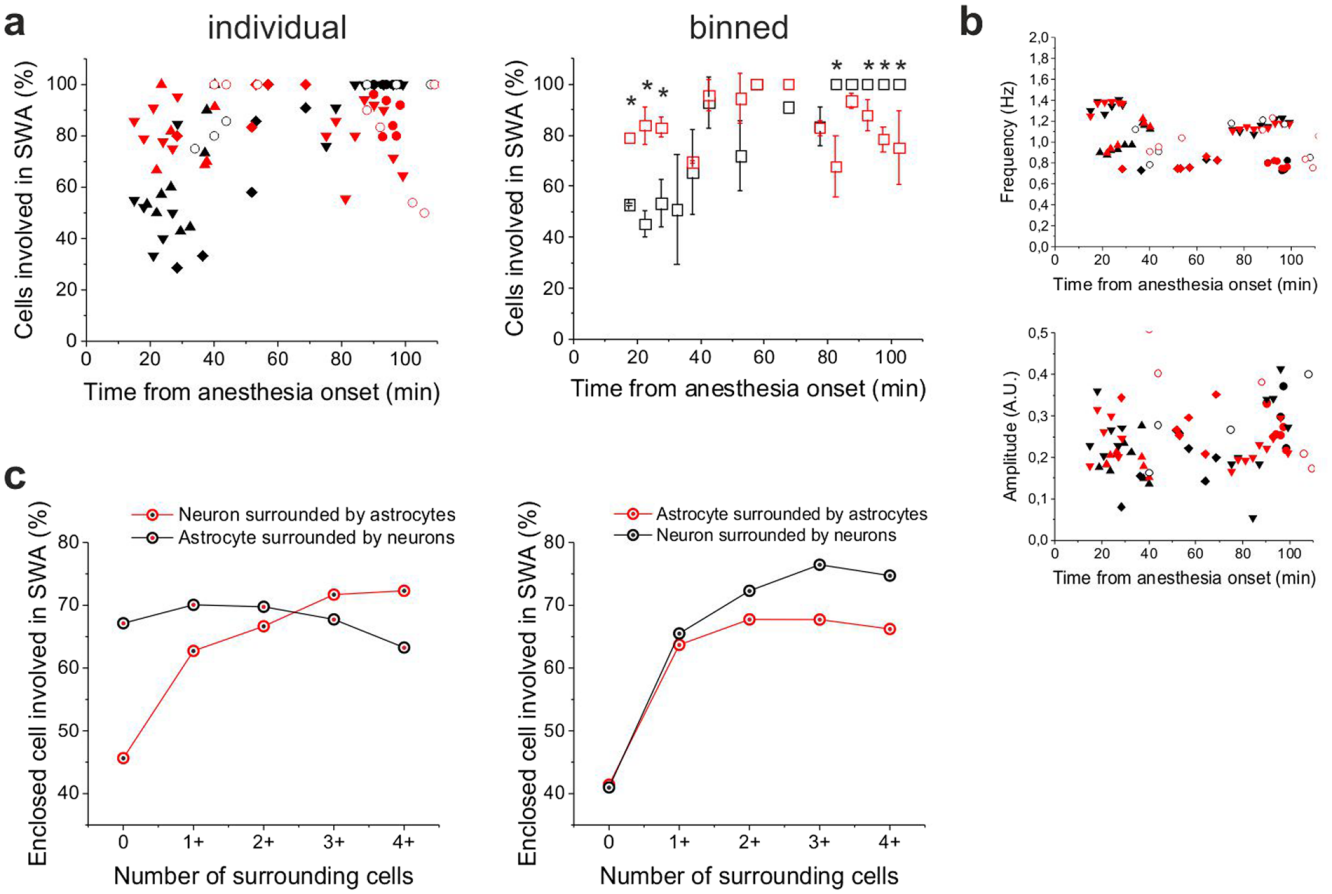
Extensive synchronized astrocytic activity contributes to the emergent neuronal synchronization in the early stage of ketamine/xylazine anesthesia. (**A**) The ratio of astrocytes (red) and neurons (black) showing repetitive, UP state-related Ca^2+^ transients in percentage of all astrocytes or neurons, respectively, in the field of view from 5 different (P21-P83) animals (*left*) and their averages at 5-minute intervals (mean ± SD) (*right*). Asterisks show significant differences between astrocytes and neurons (p < 0.001). (**B**) Frequency and amplitude of detected UP state-related Ca^2+^ transients. (**C**) Effect of the number of surrounding (d < 100 μm) active cells on the ratio of astrocytes (red) and neurons (black) showing repetitive, UP state-related Ca^2+^ transients in percentage of all astrocytes or neurons, respectively, having the same number (0) or same minimum number (1+, 2+, etc.) of adjacent cells. Percentage data are calculated from n = 5 (P21–P83) animals. Red markers represent astroglia, black markers represent neurons. Different symbols represent data from different animals. Astrocytes were identified by SR101 loading (solid symbols) or by astrocyte-specific expression of the red-fluorescence RGECO protein under the control of a GFAP promoter (empty symbols). Ketamin/xylazine was applied at t = 0.

To explore the potential interaction between neuronal and astroglial network activities and the role astrocytic activation may play in the SWA, we analyzed the temporal and spatial profiles of neuronal and astrocytic Ca^2+^ transients during the anesthesia from 15 to 100 min following ketamine/xylazine administration (Figs 4 and 5). In the temporal domain no difference was observed between the astrocytic and neuronal signals. Cross-correlation of fluorescent traces showed no time lag between the neuron-neuron (n = 1742) or astrocyte-astrocyte (n = 7514) pairs (Figs 4C and 5B) and also no temporal delay was measured between the neuronal and astrocytic transients (n = 1693, Figs 4C and 5B). Therefore, neuronal and astrocytic calcium transients are fully synchronized in the temporal domain, neither one precedes the other. It is to note, however, that short delays in the low millisecond range may exist that is beyond the temporal resolution (typically ~30 ms) of our imaging setup. In addition, no significant difference was found between the frequency of neuronal and astrocytic peaks, albeit they slightly increased within experiments starting from the onset of ketamine/xylazine application and were also moderately variable between different animals (Fig. 5B). Similarly, amplitudes of neuronal and astrocytic peaks were also indistinguishable (Fig. 5B).

In contrast to the temporal characteristics, the spatial extensions of the astrocytic and neuronal transients were clearly distinguishable: it was found that significantly more astrocytes than neurons were active in the early phase of SWA (Fig. 4A,B). To quantify this difference, we calculated the ratio of neurons and astrocytes showing recurrent activity. The number of active cells was divided by the total number of GCaMP2-expressing astrocytes or neurons in the field of view, giving the astrocytic and neuronal spatial consistency ratios (Figs 4B and 5A). Neuronal spatial consistency increased over time that was showed by the increasing ratio of cells joining the synchronized activity (Fig. 5A). After approximately 80 min of anesthesia, virtually all neurons were involved in the synchronized activity in line with previous studies showing that after 60 min, ~90% of neurons participated in synchronous firing^35^. Importantly, however, the ratio of astrocytes involved in the synchronous activity was significantly higher (p < 0.001) than that of neurons in the first 30 min of anesthesia (Figs 4A,B and 5A), arising the possibility that the astrocyte network may play a critical role in the emergence of slow neuronal oscillations during sleep. Astrocytic and neuronal spatial consistency was also found to be distinct at the late stage of anesthesia. While the neuronal network remained fully involved in the synchronized activity, astrocytic involvement declined at this stage, further suggesting that the activity of the astrocytic network may be independent from the neuronal one.

The above described divergence between the spatial consistency of neuronal and astrocytic networks was confirmed by data from 5 different rats (P21-P83, Fig. 5A). Collective data showed that spatial consistency was significantly higher in the astrocytic network than in the neuronal one (83.2 ± 3.2% vs. 50.6 ± 4.6%, p < 0.001) in the first 30 min of anesthesia. This marked difference between the spatial scale of involvement of astrocytes and neurons in the SWA may suggest that astrocytes play an important role in the build-up of synchronized activity.

Noteworthy, although SR101 is a widely used marker of astrocytes *in vivo*, to exclude the misidentification of astrocytes due to potential neuronal uptake of SR101, a transgenic rat expressing the red-fluorescent RGECO protein specifically in astrocytes under the control of a GFAP promoter was also studied (Supplementary Fig. 2). The percentage of active astrocytes and neurons as well as the amplitude and frequency of the associated Ca^2+^ transients from this rat (empty symbols in Fig. 5A,B) are in accordance with the data from the SR101-labeled animals.

The higher ratio of astrocytes involved in the early phase of SWA does not necessarily constitute causal relationship between astrocytic and neuronal activity. The emergence of wide-scale neuronal involvement in the synchronized activity might also be attributable to independent factors. To investigate whether astrocytic network activation does trigger the incorporation of additional neurons into SWA we further investigated the spatial correlation of the activity of the two networks. We found that the number of active astrocytes (showing SWA-related activity) in the vicinity of a neuron significantly influences whether the given neuron is getting involved in SWA (Fig. 5C). Only 46% of neurons not having an active astrocyte within 100 µm were found to be active. In contrast, 72% of neurons having at least four active astrocyte neighbours within the same radius showed SWA-related activity. This correlation suggests that the activity of neurons and astrocytes are correlated, but it does not contain information about which cell is activating the other. The higher number of active astrocytes in the vicinity of an active neuron may also reflect the activation of astrocytes by nearby active neurons. However, in this case, astrocytic activity should also be increased when the number of active surrounding neurons is increased. However, this correlation was opposed by the experimental data, showing that the number of nearby active neurons does not affect the activity of astrocytes (Fig. 5C). Noteworthy, the activity of neurons or astrocytes were found to be correlating to the number of nearby neurons or astrocytes, respectively, confirming the ability of intra-network synchronization in both cell populations (Fig. 5C).

### Inhibition of astrocytic synchronization suppresses slow wave activity

**Figure 6:**
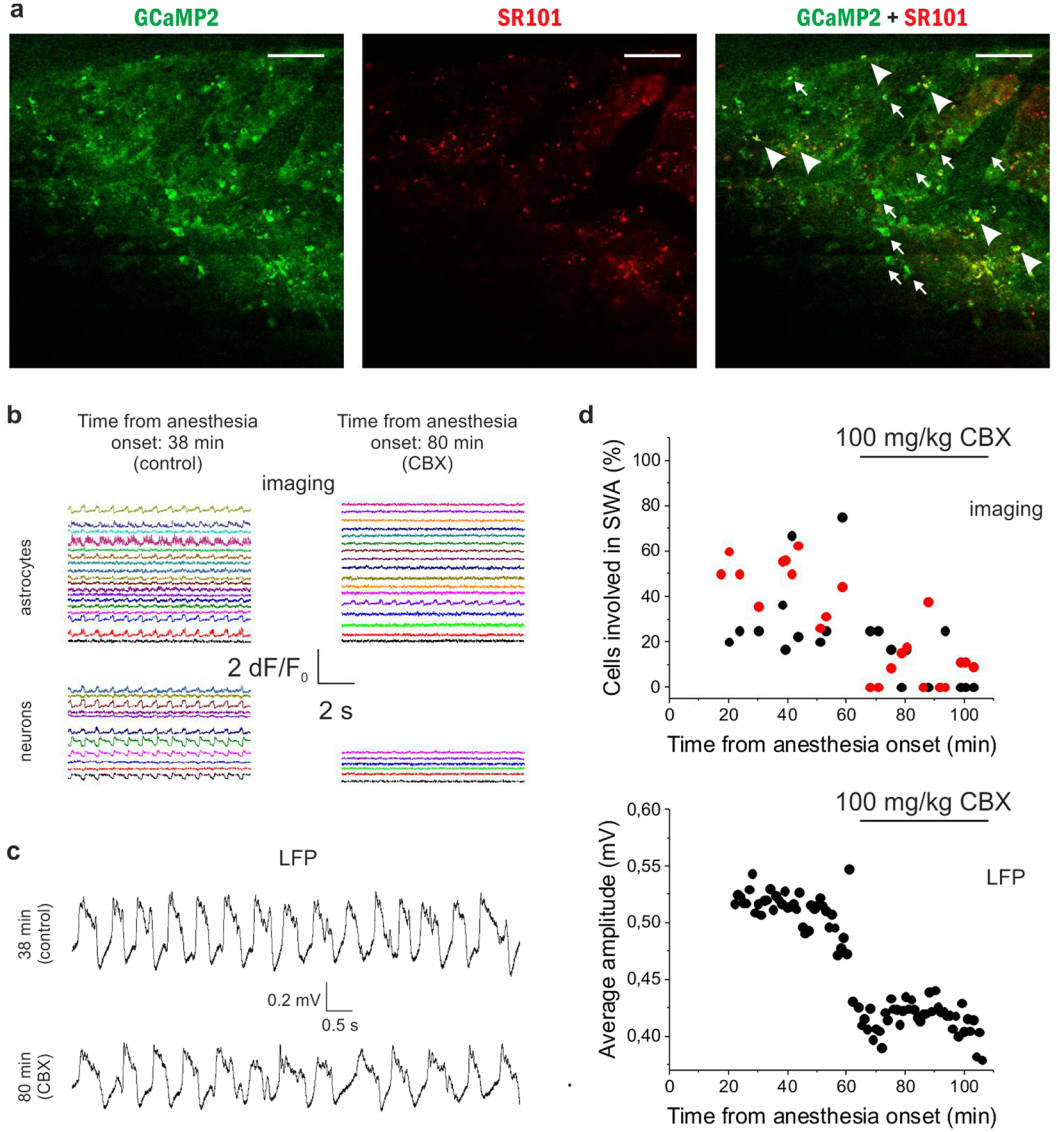
The gap junction blocker carbenoxolone (CBX) reduces the number of astrocytes and neurons taking part in the slow wave-associated network activity. (**A**) Expression of GCaMP2 (green), labeling of astrocytes with SR101 (red). Both neurons (some marked by arrows) and astrocytes (some marked by arrowheads) express GCaMP2. Scale bars: 100 µm (**B**) *Left:* 10-sec segments of ∆F/F_0_ fluorescent intensity traces of all identified astrocytes (n = 18) and neurons (n = 11) in the imaged area in the absence of CBX at 38 min following anesthesia. *Right:* 10-sec segments of ∆F/F_0_ fluorescent intensity traces of all identified astrocytes (n = 17) and neurons (n = 6) in the imaged area in the presence of CBX at 80 min following anesthesia. Different cells were imaged at 38 and 80 min. (**C**) Representative LFP recordings low-pass filtered at 20 Hz at different stages shown in B. (**D**) *Top*: Ratio of V1 neurons (black) and astrocytes (red) in the field of view showing repetitive Ca^2+^ transients under ketamine/xylazine anesthesia before and after i.p. administration of 100 mg/kg CBX. Individual markers represent data from each 60-sec imaging sessions. *Bottom*: Average amplitude of local field potential (LFP) positive deflections in each 1-minute segment of the LFP recording in the rat primary visual cortex V1 before and after i.p. administration of 100 mg/kg CBX. All data were recorded from the V1 area of a P44 rat (n = 24 imaging session, 60 s each).

Since astrocytic gap junctions are far more abundant than neuronal ones^36^ and through them the astrocytic syncytium can be quickly synchronized in large areas, the earlier extensive synchronization of the astrocytic network suggested the role of gap junctions in the build-up of SWA. To explore this possibility, we blocked gap junctional communication, thereby inhibiting the spreading of Ca^2+^ transients throughout the astrocytic syncytium. Neuronal and astrocytic Ca^2+^ dynamics as well as LFP in layer 2 of the V1 area were monitored for 60 min during anesthesia, followed by i.p. application of the gap junction inhibitor carbenoxolone (CBX) at 100 mg/kg concentration that was demonstrated to be effective in blocking gap junctions *in vivo* ^37, 38^. We observed that the ratio of astrocytes showing periodic Ca^2+^ transients significantly decreased after CBX application (Fig. 6A). In average, 47.4 ± 3.6% of astrocytes were active during the control period compared to 9.1 ± 3.2% after CBX treatment (p < 0.001). In parallel, the ratio of neurons involved in the slow wave-related Ca^2+^ activity was also significantly decreased (from 34.7 ± 6.1% to 9.0 ± 3.3%, p = 0.001), suggesting that astrocytic synchronization plays a causal role in the generation and/or maintenance of neuronal synchrony.

Importantly, the inhibitory effect of CBX was also evident on the simultaneously measured LFP. In parallel with the decreasing ratio of Ca^2+^ spiking neurons, the amplitude of the positive deflections of the LFP signal was significantly decreased (Fig. 6B, 0.512 ± 0.002 mV *vs*. 0.416 ± 0.002 mV during the control period and CBX application, respectively, p < 0.001), confirming that decreasing number of neurons took part in the synchronized activity after CBX application.

**Figure 7:**
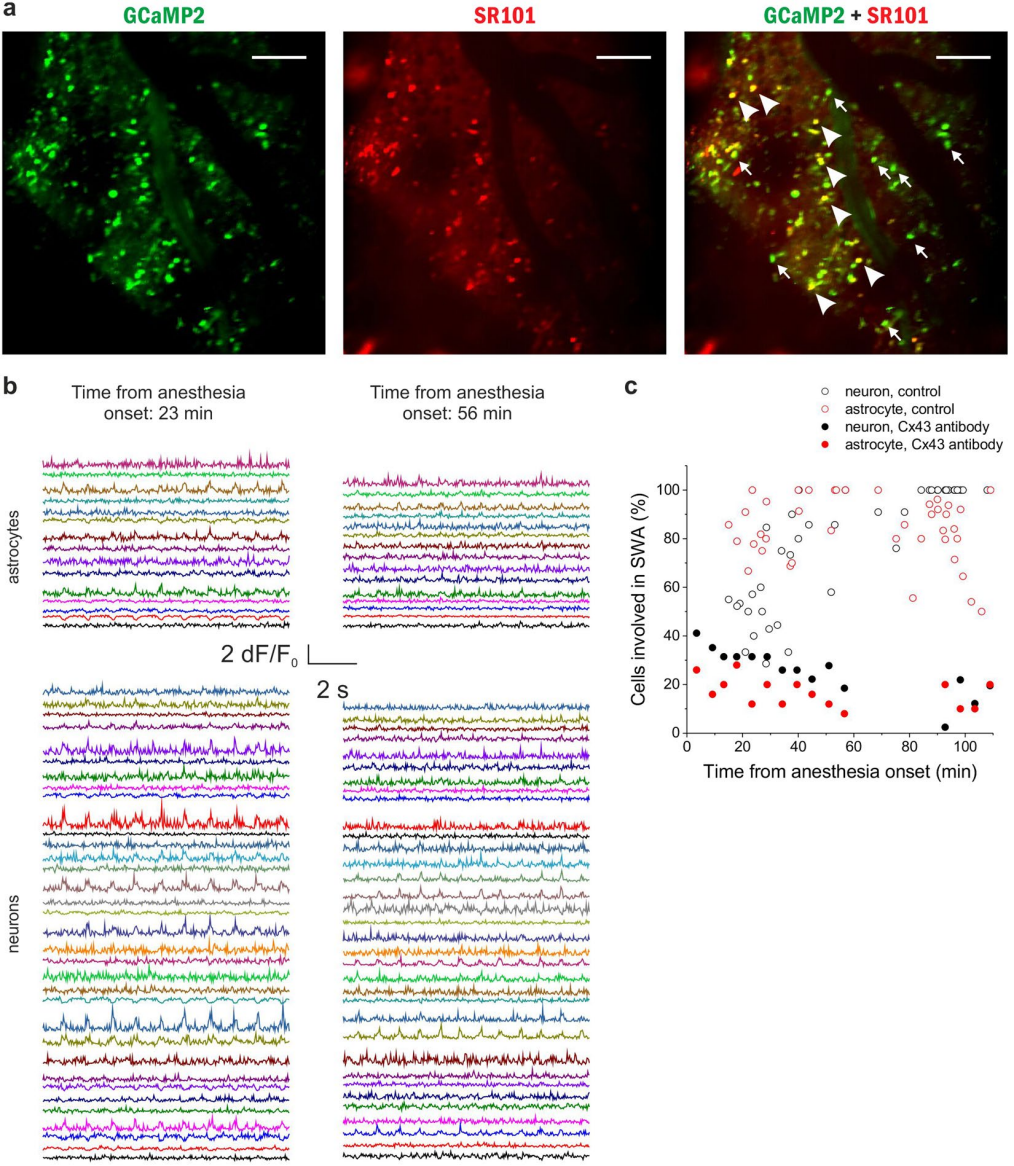
Specific blockade of the astrocytic gap junction subtype Cx43 inhibits synchronization in both astrocytes and neurons *in vivo*. (**A**) Expression of GCaMP2 (green), labeling of astrocytes with SR101 (red). Both neurons (some marked by arrows) and astrocytes (some marked by arrowheads) express GCaMP2. Scale bars: 100 µm (**B**) *Left:* 10-sec segments of ∆F/F_0_ fluorescent intensity traces of all identified astrocytes (n = 15) and neurons (n = 34) in the imaged area at 23 min following anesthesia. *Right:* 10-sec segments of ∆F/F_0_ fluorescent intensity traces of all identified astrocytes (n = 15) and neurons (n = 34) in the imaged area at 56 min following anesthesia. The same cells were imaged at 23 and 56 min. Cx43 antibody was applied 24 hours before the experiment. (**C**) The ratio of astrocytes (red) and neurons (black) showing repetitive, UP state-related Ca^2+^ transients in percentage of all astrocytes or neurons, respectively, in the field of views in the same P43 rat in the presence of Cx43 antibody (solid markers).Individual markers represent data from each 300-sec imaging sessions. Empty markers represent the ratio of astrocytes (red) and neurons (black) showing repetitive, UP state-related Ca^2+^ transients in the absence of Cx43 antibody. Note that large-scale synchronization is inhibited by the Cx43 antibody in both neurons and astrocytes. All data were recorded from the V1 area of a P43 rat (n = 15 imaging session, 300 s each).

Although CBX is a widely used inhibitor of gap junctional communication, and, to our knowledge, the only gap junction inhibitor that can penetrate the blood-brain barrier, it is not specific to astrocytic gap junctions, but it also acts on neuronal connexins^39, 40^ and cardiac gap junctions^41^. Since inhibition of neuronal Cx36 may also result in reduced synchronization^42^, we opted to specifically block the astrocytic Cx43 isoform with a specific antibody against its gating peptide segment^43^, which has previously been demonstrated to inhibit Cx43 function^16, 39^. The Cx43 antibody (0.75 mg/ml, 10 μl) was applied to the cortical surface 24 hours before the experiment to ensure antibody penetration into layer 2. Although SWA-related Ca^2+^ transients could still be observed in the presence of the Cx43 antibody, the ratio of both neurons and astrocytes involved in the rhythmic activity was significantly reduced (Fig. 7). Following ketamine/xylazine administration, the increase in the number of neurons and astrocytes participating in SWA was not observed. Instead, the ratio of active cells slowly decreased over time. Only 26.2 ± 1.8% of neurons and 14.3 ± 1.7% of astrocytes showed SWA-related activity at 20 to 60 min of anesthesia in the presence of Cx43 antibody compared to 63.6 ± 5.2% and 87.1 ± 3.0% in control conditions (p < 0.001 for both neurons and astrocytes). The results clearly demonstrate that specific blockade of astrocytic gap junctions inhibited SWA-related activity to extend over larger cell populations.

**Figure 8:**
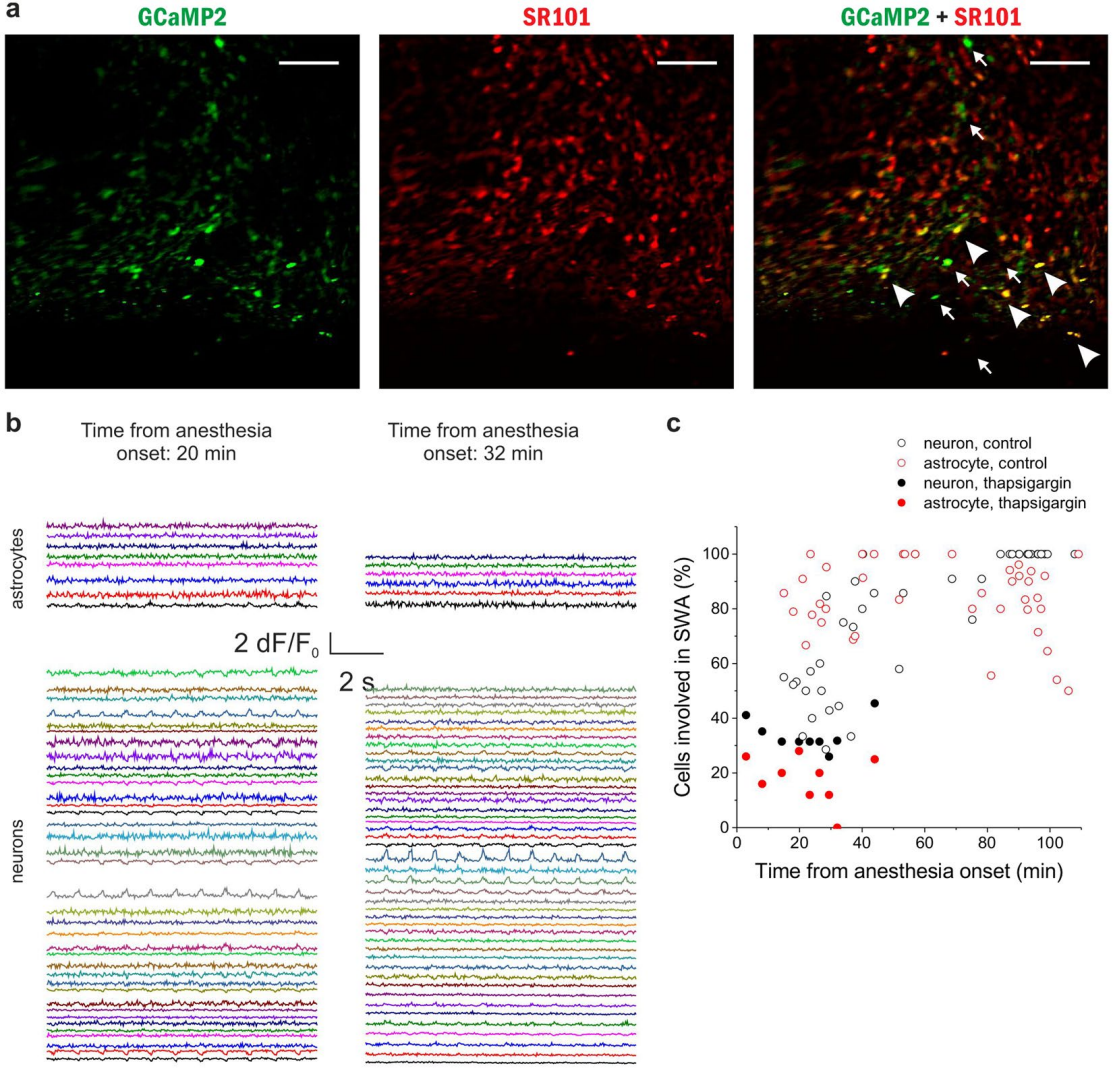
Specific reduction of the astrocytic Ca^2+^ transients by thapsigargin inhibits synchronization in both astrocytes and neurons *in vivo*. (**A**) Expression of GCaMP2 (green), labeling of astrocytes with SR101 (red). Both neurons (some marked by arrows) and astrocytes (some marked by arrowheads) express GCaMP2. Scale bars: 100 µm (**B**) *Left*: 10-sec segments of ∆F/F_0_ fluorescent intensity traces of all identified astrocytes (n = 8) and neurons (n = 37) in the imaged area at 20 min following anesthesia. *Right:* 10-sec segments of ∆F/F_0_ fluorescent intensity traces of all identified astrocytes (n = 6) and neurons (n = 46) in the imaged area at 32 min following anesthesia. Different cells were imaged at 20 and 32 min. Thapsigargin was applied 30 min before the experiment. (**C**) The ratio of astrocytes (red) and neurons (black) showing repetitive, UP state-related Ca^2+^ transients in percentage of all astrocytes or neurons, respectively, in the field of view of the same P38 rat in the presence of thapsigargin (solid markers). Individual markers represent data from each 300-sec imaging sessions. Empty markers represent the ratio of astrocytes (red) and neurons (black) showing repetitive, UP state-related Ca^2+^ transients in the absence of thapsigargin. Note that large-scale synchronization is inhibited by thapsigargin in both neurons and astrocytes. All data were recorded from the V1 area of a P38 rat (n = 9 imaging session, 300 s each).

To further explore the causal relationship between astrocytic activity and SWA, we used thapsigargin, a specific inhibitor of endoplasmic reticulum Ca^2+^-ATPases, that prevents refilling of internal Ca^2+^ stores and was demonstrated to specifically inhibit astrocytic Ca^2+^ transients *in vivo*, while leaving neuronal Ca^2+^ signals unaffected^44^. Thapsigargin (0.5 mM, 10 μl) was applied to the cortical surface 30 min before the experiment. Thapsigargin reduced the ratio of active neurons and astrocytes (Fig. 8) to an extent similar to CBX and Cx43 antibody (32.9 ± 2.7% of neurons and 16.2 ± 4.2% of astrocytes, p < 0.001 compared to control for both neurons and astrocytes). Also, similar to the Cx43 antibody (Fig. 7), the ratio of active cells slowly decreased over time, opposite to the increase observed in untreated animals (Fig. 5A).

All three investigated compounds, inhibiting intercellular Ca^2+^ spreading in astrocytes decreased the astrocytic and neuronal involvement in SWA, conclusively suggesting that astrocytes play a causal role in the generation and/or propagation of SWA.

## Discussion

Despite the widely recognized relevance of cortical SWA, its cellular mechanism is currently far from being understood. The experiments described in this paper demonstrate, for the first time, that the astrocytic network does display extensive synchronized activity during *in vivo* SWA, simultaneously with the activation of the neuronal network. Using a rat line stably expressing the genetically encoded calcium sensor protein GCaMP2 in astrocytes and interneurons, we showed that in the ketamine/xylazine anesthesia model of slow-wave sleep, large fractions of both cell types are concurrently activated during the UP states in the superficial layers of the primary visual cortex. Temporal parameters, such as frequency of the observed calcium transients were not significantly different in the two cell types. Cross-correlation analyses also did not show any time lag between the fluorescent traces, suggesting a functional link between their activities. In the spatial dimension, however, significant differences were observed between the astrocytic and neuronal activity. Importantly, the ratio of active astrocytes was significantly higher than that of active neurons in the first 30 min of anesthesia, suggesting a principal role of astrocytes in the generation or spreading of SWA. After about 60 min virtually all neurons and astrocytes participated in the synchronous oscillatory activity, followed by a steep decline in the spatial consistency of the astrocytic calcium signals, while the neuronal synchronization still involved all neurons. This difference may further strengthen the view that astrocytic activity during SWA is not a response to neuronal firing, rather an independent and potentially causal actor. Since a major route of astrocytic synchronization is mediated by gap junctions^45^, we addressed this potential role by applying the general gap junction inhibitor CBX and an inhibitory antibody specific to astrocytic Cx43 connexins. The purpose of these agents was to prevent physical coupling of astrocytes and to reduce their capacity for long-range synchronization. We demonstrated that both CBX and Cx43 antibody application significantly reduced the involvement of not only astrocytes, but also neurons in SWA, suggesting that astrocytes play a causal role in SWA. Furthermore, CBX also reduced the amplitude of LFP deflections, confirming that blockade of direct coupling between astrocytes significantly reduced the number of neurons taking part in SWA. Importantly, perturbation of long-range astrocytic synchronization by another mechanism, through the astrocyte-specific inhibition of intracellular Ca^2+^ transients by thapsigargin provoked the same reduction in SWA-related astrocytic and neuronal activity. The significant reduction of neuronal SWA following specific inhibition of astrocytic network synchronization by two pathways makes causal role of astrocytes in SWA generation the most reasonable explanation of the results.

### Both astrocytes and interneurons are powerful modulators of neuronal excitability

Computational studies suggested various molecular and cellular mechanisms as generators of UP states in slow-wave sleep. According to these neurocentric explanations, the emergence of synchronous neuronal activity may be the consequence of the amplification of miniature synaptic potentials by intrinsically bursting neurons^46^ or spontaneous activation of a small population of neurons^47^. The common property of these models is that the initial expansion of synchronous firing from a few cells to a smaller network requires the balancing of excitation by inhibitory currents. This feature makes GABA*ergic* interneurons, that control the excitability of pyramidal cells at multiple subcellular regions^48^, key candidates for orchestrating neuronal activity. Indeed, GABA_A_ receptor mediated synaptic currents have been shown to lie behind the emergence of gamma oscillations^49^. Recently, it was also demonstrated that GABAergic interneurons are specifically activated during sleep in the cortex of both human^50^ and rodents^51, 52^, further supporting their contribution to SWA generation.

Importantly, however, interneurons are not the only cell types that can provide synchronized inhibition over a large number of neurons. Astrocytes are also endowed to control the excitability of nearby neurons (both interneurons and pyramidal cells) and to spread information through their heavily interconnected network, thereby establishing neuronal synchronization on large spatial scales^53^. Many of the essential features of astrocytes and astrocytic networks, like bidirectional communication with neurons^23^, covering of up to millions of synapses^54^ and their extensive physical interconnection by gap junctions, position them as key players in a wide range of physiological functions from brain rhythm generation^15^, through memory formation^55^ up to behavior^2^.

### Astrocytic network activation contributes to the build-up of neuronal synchronization

There is a consensus that DOWN states during SWA are commenced by the termination of an excitatory drive^56, 57^, rather than by the emergence of an active inhibition. This view is strongly supported by the fact that inhibitory interneurons are also inactive in the DOWN states. Astrocytes are able to exert long-lasting inhibitory control over neurons by increasing the ambient extracellular GABA concentration and by generating tonic inhibition^8–10, 58, 59^. Therefore, in principle, they may function in the opposite way, providing sustained inhibition during the DOWN states. This effect may have been easily overlooked in previous studies since astrocytes do not experience large membrane potential fluctuations, therefore they do not contribute to the widely studied electrophysiological signals to a significant extent.

In the present *in vivo* study, however, we observed that, just like neurons, astrocytes are active in the UP states. Thus, they may take part in the initiation rather than the termination of neuronal synchronization. A small number of pioneering *in vitro*, single-cell studies also support this finding. Electrophysiological double recording of neurons and astrocytes revealed that the two cell types display coherent activities during slow-wave oscillations *in vitro* ^53^. Importantly, the same study also demonstrated that the beginning of neuronal DOWN states was preceded by glial hyperpolarization, suggesting causal relationship^53^. In another study, stimulation of single astrocytes was found to trigger neuronal UP states^60^. The latter study also revealed that disruption of astrocytic Ca^2+^ wave propagation by injection of a Ca^2+^ chelator into a single astrocyte inhibited both spontaneous and stimulated UP states. Chelation of calcium in a single astrocyte was also shown to inhibit long-term potentiation specifically in the domain of the chelator-loaded astrocyte^61^. The universality of this mechanism is further illustrated by the fact that astrocytic calcium chelation also produced neuronal network desynchronization in the hippocampus *in vitro* ^62^.

Recently, an *in vivo* study by Poskanzer and Yuste^63^ described observations supporting *in vitro* results. In urethane-anesthetized mice, the authors described spontaneously alternating recurrent cortical states of synchronized activity, representing a notable model, midway from *in vitro* preparations generating sparse local field potential activity in every 5.15 ± 0.27 min^60^ to physiological ~1 Hz SWA (present study). In their *in vivo* study, the authors showed that prior to the synchronized states occurring every 5.4 ± 0.37 min (0.003 Hz), astrocytic Ca^2+^ transients with a mean duration of 15.7 ± 0.83 s occurred, implying causality between the two events. Findings reported in the present study underlie this hypothesis by demonstrating that astrocytes actively contribute to the synchronization of neuronal networks not only in less complex *in vitro* preparations^53, 60–62^ and during sparsely occurring synchronized states *in vivo* ^63^, but also during genuine SWA (~1 Hz), a complex, wide-scale, fundamental physiological phenomenon. Importantly, taking advantage of our high-speed two-photon acquisition system (30–156 Hz sampling rate), we were able to detect fast Ca^2+^ signals with duration of a few hundred milliseconds that were not resolved in the above study using 1–4 Hz sampling rate^63^. Unlike the slow Ca^2+^ transients that showed no apparent synchronization^63^, the fast Ca^2+^ signals revealed in our experiments were highly synchronized both among astrocytes and co-synchronized to neurons. This might be indicative of the fact that the bidirectional communication at the neuron-glia interface operates faster than expected. However, despite the fundamental difference in the dynamics of the slow^63^ and fast (present study) Ca^2+^ transients, both signals were shown to contribute to the emergence of neuronal synchronization. The coupling of both slow and fast Ca^2+^ signalling to the neuronal activation may be indicative of the scale-free nature of the astrocytic Ca^2+^ dynamics^64^, featuring fractal organization of neural functions operating at time-scales with orders of magnitude disparity^65^. Although model differences might also be accounted for the phenomena, universality of neocortical coupling of astrocytic and neuronal circuits may highlight a role for system adaptation.

Importantly, the significant impact of the number of active neighbouring astrocytes on the involvement of a given neuron in the SWA ﻿(Fig. 5C) strongly highlights the causal role the astrocytic network may play in the generation of neuronal synchronization. The importance of neighbouring astrocyte density may correspond to the domain-like control that astrocytes exert on neuronal excitability. In contrast to neurons that send and receive connections to and from long distances by their axons and dendritic trees, astrocytes occupy local, non-overlapping domains^66^. The efficacy of all synapses within a domain is controlled by a single astrocyte that can release both inhibitory (e.g. GABA, ATP) and excitatory (e.g Glu, d-serine) signalling molecules^8, 9, 59, 61, 67–71^. Since the dendritic tree of a neuron is covered by multiple astrocytes, increasing the number of neighbouring active astrocytes around a neuron should increase the strength of astrocytic control. A theoretical modelling study by Savtchenko and Rusakov^72^ previously showed that spatially clustered (i.e. domain-like) control over neuronal excitability is more efficient in generating and maintaining rhythmic activity than neuron-like spatially distributed inputs. Based on their modelling results the authors proposed that astrocytes are designed to preserve network oscillation^72^. This hypothesis was reinforced by a recent modelling study showing that intercellular Ca^2+^ signalling potentially can introduce slow oscillation in neurons^73^. Our experimental data strongly supports this hypothesis by demonstrating that increasing astrocytic influence on neurons indeed drives them to join the oscillatory activity (Fig. 5C). In this context it is also important to note, that the ratio of astrocytes involved in the SWA was found to start decreasing right after virtually all neurons joined the simultaneous activity (Figs 4 and 5). This observation further supports the view that astrocytic activity corresponds to the generation or maintenance, rather than termination of SWA.

Therefore, combining the results of previous *in vitro* and *in vivo* experiments as well as theoretical studies with the present experimental *in vivo* findings, it is safe to conclude that the astrocytic network plays a role in the build-up phase of synchronous neuronal activity and that active contribution of astrocytes to the synchronization of the neuronal network is a viable hypothesis.

### Causal role of astrocytes in SWA generation

It is a fundamental question what is the relationship in the observed concurrent activation of neurons and astrocytes. Whether astrocytic activity merely follows neuronal firing or do astrocytes actively contribute to the generation or spreading of neuronal synchronized activity? Two considerable experimental limitations prevented us from directly targeting this issue. First, the activity of pyramidal cells, an important cell type in all kinds of cortical activity could not be monitored due to the lack of GCaMP2 expression in these cells. Although LFP recordings gave information about the activity of pyramidal cells as well, our tools were not amenable to record their cellular-level spatial activity patterns directly. Second, the first 10–15 min of anesthesia could not be recorded, since this time was required to position the anesthetized animal in the microscope and to set up electrophysiological and imaging apparatus. Therefore, to address the question of causality, we perturbed astrocytic synchronization beforehand and measured whether or not neuronal SWA is affected by this intervention. Astrocytic synchronization was targeted by blocking gap junctional communication or inhibiting intracellular Ca^2+^ transients.

Gap junctions are the major path through which astrocytes can be synchronized^45^. However, gap junctions are expressed not only on astrocytes, but also on interneurons^74, 75^. Unfortunately, no specific small molecule inhibitor of either the glial or neuronal form of the gap junction forming connexin hemichannels exists. Available specific mimetic peptides, like the astrocyte-specific Cx43 inhibitor Gap19 cross the blood-brain barrier (BBB) to a very small extent even when combined with TAT sequence^36^, therefore they cannot be applied systemically. The small molecule inhibitor CBX that cross the BBB, however, is not selective, thus it inhibits both neuronal and astrocytic gap junctions. Hence, we applied both approaches, the general gap junction inhibitor CBX i.p. and a specific inhibitor of astrocytic gap junctions, a Cx43 antibody locally.

Compared to the control SWA period, application of CBX significantly reduced the ratio of astrocytes and neurons displaying Ca^2+^ transients corresponding to UP states. In parallel, the LFP amplitude was also considerably reduced, demonstrating that gap junctions do play a significant role in SWA generation or maintenance. Since activity of the non-imaged pyramidal cells is a major contributor to the LFP signal, the reduction in LFP amplitude suggests that not only interneuronal, but also the pyramidal activity is affected by CBX application. Due to the non-specificity of CBX, however, we could not unambiguously conclude whether the observed changes are due to the blockade of the astrocytic or neuronal isoforms. Therefore, the astrocytic Cx43 isoform was specifically blocked by a Cx43 antibody that was previously successfully applied *in vitro* for specific astrocytic gap junction inhibition^16, 39^. However, since this antibody does not cross the BBB and the spatial extent of the affected area upon local pressure injection cannot be reliably controlled, the Cx43 antibody could not be applied during the experiment. Instead, astrocytic and neuronal activity measured in its presence was compared to activities obtained in the absence of the inhibitor. The significant reduction of neuronal SWA activity in the presence of the specific Cx43 antibody suggests that astrocytic gap junctions do contribute to SWA generation or spreading. This contribution is further supported by the *in vitro* observation that astrocytic connexin 43 hemichannel opening increases the amplitude of UP states and neuronal firing in rats^76^. To resolve whether the critical contribution of astrocytes is required for the SWA generation or spatial extension of the synchronized activity will require further studies targeting the wakefulness-sleep and sleep-wakefulness transitions.

Finally, the causal role of astrocytes in the generation or spreading of SWA is further strengthened by the fact that astrocyte-specific inhibition of intracellular Ca^2+^ transients by thapsigargin also significantly reduced the ratio of neurons involved in SWA. The observed effect in the presence of thapsigargin clearly excludes the possibility that astrocytic gap junction blockade achieves neuronal SWA reduction by a mechanism other than astrocytic Ca^2+^ signalling.

### Contribution of astrocytes to other types of neuronal synchronization

It has been demonstrated that specific inhibition of astrocytic vesicular release leads to significant reduction of gamma oscillations and consequently impairs recognition memory in mice^77^, further highlighting the role astrocytic networks play in higher cognitive functions. Contribution of astrocytic networks is also supported by recent computational studies that showed astrocytic tuning of hippocampal neuronal network activity in the delta frequency range^15^. The same study suggested the possibility of astrocytic background in the emergence of epileptic activity, which we previously confirmed by demonstrating that astrocytic and neuronal networks show concurrent, synchronized activity and that disruption of astrocytic synchronization by CBX or Cx43 antibody prevents the generation of seizure-like events^16^. These data indicate that astrocytic activity is not merely coupled to neuronal firing pattern during brain oscillations, but it may play a causal role in triggering rhythmic neuronal activity. Our transgenic rat line stably expressing GCaMP2 in both astrocytes and neurons may serve as a model to study such higher cognitive functions in rat that clearly outperforms the widely used mouse models in cognitive tasks^78^.

In conclusion, multiple lines of *in vitro* experimental data suggest that astrocytic networks are able to fundamentally control neuronal network activity and significantly contribute to the generation or spreading of brain oscillations. In line with these *in vitro* findings, our *in vivo* data demonstrate, for the first time, that astrocytic networks display coherent activity during SWA and this activity is proposed to play a causal role in SWA generation. We envisage that the *in vivo* identification of the astrocytic network as a key player in brain oscillations will deepen our understanding of these fundamental features of the brain function.

## Methods

### Animals

All animal procedures were performed according to standard ethical guidelines and approved by the local Animal Care Committee and the Government Office for Pest County (reference numbers PEI/001/3671-4/2015 and 22.1/2727/3/2011). The transgenic rat strain stably expressing the CAG-GCaMP2 transgene was established earlier using the *Sleeping Beauty* transposon based gene delivery technology^21^. Additionally, two other homozygous rat strains were established with the same transposon technology: one expressing the red fluorescent RGECO calcium indicator under the regulation of the astrocyte specific GFAP promoter; the other strain containing both the CAG-GCaMP2 and the rGFAP-RGECO transgenes in a homozygous (one haploid copy) form was established by the careful selection of the progenies from crossing the two original homozygous transgenic strains. *In vivo* experiments were performed on 4–8 week old transgenic male and female Wistar rats that were housed in a 12 hour light/dark cycle. All drugs were obtained from Sigma-Aldrich unless stated otherwise.

### Slice culture

Since expression of GCaMP2 was largely reduced in acute slice preparations^21^ we used organotypic hippocampal slice cultures prepared according to the method of Kovács *et al*.^79^, with minor modifications. Briefly, 380 μm thick hippocampal slices were cut from 6-day-old Wistar rats using a McIlwain tissue chopper, and separated in ice-cold Gey’s balanced salt solution (GBSS) composed of (mM): 33 glucose, 2 CaCl_2_, 1.03 MgCl_2_, 0.28 MgSO_4_, 0.22 KH_2_PO_4_, 27.02 NaHCO_3_, 120 NaCl, 0.84 Na_2_HPO_4_, 5 KCl, 10 HEPES, pH 7.2. The slices were placed on a culture plate insert (MilliCell-CM, Merck Millipore, Budapest, Hungary) and the inserts were transferred to a 6-well culture plate. Each well contained 1 ml of culture medium consisting of 50% Opti-MEM® I Reduced Serum Medium (Life Technologies, Budapest, Hungary), 25% HBSS, 25% horse serum, D-glucose (0.25 g/ml) and gentamicin (0.1 mg/ml). The organotypic cultures were maintained in a humidified incubator bubbled with a 5% CO_2_/95% O_2_ atmosphere at 37 °C for 3 days. On day 4, culture medium was replaced with another formulation and used afterwards (76% Neurobasal®-A Medium, minus phenol red (Life Technologies), 1% GlutaMAX® Supplement, 2% B-27® Supplement, serum free, 1% D-glucose, 20% horse serum supplemented with (mM, final concentration): 0.1 D,L-α-Tocopherol, 0.5 ascorbic acid, 0.025 β-mercapto-ethanol; 0.1 mg/ml gentamicin). Culture medium was changed twice a week. Cultures were used for analyses within 3 weeks.

### Immunohistochemistry

GAD67/GCaMP2 and GFAP/GCaMP2 colocalization studies were performed using a slightly modified protocol of Weiswald *et al*.^80^. Briefly, organotypic cultures were fixed in 4% paraformaldehyde +1% Triton X-100 for 3 h at 4 °C. Slices were washed and then further fixed sequentially in 50%, 90% and 50% methanol for 5 min at −20 °C. Cultures were washed in PBS, blocked with 1% bovine serum albumin +5% goat serum +0.1% Triton X-100 for 1 h at 4 °C. Rabbit anti-GFP (1:500, ab290, Abcam), mouse anti-GFAP (1:200, sc-33673, Santa Cruz) and mouse anti-GAD67 (1:1000, MAB5406, Merck Millipore) were dissolved in the blocking solution and the slices were incubated for 2 h at room temperature on a rocker shaker. After incubation in primary antibodies, the sections were washed, and incubated in goat anti-rabbit IgG Alexa Fluor 488 and goat anti-mouse IgG Alexa Fluor 546 secondary antibodies (1:200; Life Technologies) for 4 h at room temperature. Finally, the nuclei were stained with 2 µM Hoechst 33342 (10 min, RT) and the slices were covered with ProLong® Gold (Life Technologies).

To investigate NeuN/GCaMP2 and Iba1/GCaMP2 colocalization, organotypic cultures were fixed in 4% paraformaldehyde, washed, and pretreated with 3% hydrogen peroxide for 10 min followed by 1% bovine serum albumin in PB containing 0.5% Triton X-100 for 30 min at room temperature. Then, the cultures were placed in goat anti-GFP (1:1000, ab5450, Abcam) for 24 h at room temperature. After incubation in primary antiserum, the sections were washed and incubated in biotinylated donkey anti-goat secondary antibody (1:1000; Jackson ImmunoResearch, West Grove, PA) for 1 h followed by washes and incubation in avidin-biotin-horseradish peroxidase complex (1:500; Vector Laboratories) for 1 h. Subsequently, sections were treated with FITC-tyramide (1:8000) and H_2_O_2_ in Tris hydrochloride buffer (0.1 M, pH = 8.0) for 6 min. Then, mouse anti-NeuN as a marker of neuronal nuclei (1:500; Millipore, Billerica, MA, cat. number: MAB377) or rabbit anti-ionized calcium-binding adapter molecule 1 (Iba1) as a marker of microglial cells (1:1000; Wako, cat. number: 019–197419) was applied at room temperature overnight. Sections were then incubated in donkey Alexa Fluor 594 anti-mouse secondary antibody (Life Technologies, Grand Island, NY) for 2 h. Finally, the sections were mounted, dehydrated and coverslipped with Cytoseal 60 (Stephens Scientific, Riverdale, NJ).

### *In vitro* imaging

Slice cultures were transferred to the recording chamber mounted on an upright microscope (Olympus BX61WI) equipped with a FluoView300 confocal laser-scanning system (Olympus, Tokyo, Japan) or on a two-photon microscope (Femto2D, Femtonics, Hungary) using a 40× water immersion objective (N.A. 0.80). Slices were superfused with oxygenated (95% O_2_, 5% CO_2_) artificial cerebrospinal fluid (ACSF) (5 ml/min, 30 °C), containing (in mM): 129 NaCl; 1.23 NaH_2_PO_4_; 10 glucose; 1.6 CaCl_2_; 3 KCl; 21 NaHCO_3_; 1.8 mM MgSO_4_ (pH 7.4). In confocal microscopy GCaMP2 fluorescence was excited at 488 nm and emission was detected between 510–530 nm. In two-photon microscopy GCaMP2 was excited at 900 nm with a MaiTai femtosecond laser source (Spectra Physics, Santa Clara, CA, USA) and the emitted fluorescence was monitored at 475–575 nm.

### *In vitro* electrophysiological recordings

Whole-cell recordings in organotypic slices were made at ∼32 °C from cells identified under continuous GCaMP2 imaging. Borosilicate glass capillaries had open-tip resistances between 4–6 MΩ. For current clamp recordings the intracellular solution contained (in mM): 135 K-gluconate, 10 HEPES, 10 Na-phosphocreatine, 4 KCl, 4 Mg-ATP, 0.3 Na-GTP, pH 7.2 (adjusted with KOH). Signals were recorded with Multiclamp 700A amplifiers (Axon Instruments, Foster City, CA, USA), sampled at 10 kHz (Digidata 1320 A, Axon Instruments), and stored on a personal computer for subsequent analysis. Liquid junction potential of −15.7 mV was calculated by the Clampex software and used to determine cell membrane potentials. Action potentials were evoked by somatic current injection (0.5–1.0 nA, 2 ms). Glutamate (100 μM), ATP (1 mM) and ionomycin (10 μM) were applied in perfusion bath.

### Cranial window implantation

Rats were anesthetized with ketamine-xylazine (ketamine 100 mg/kg, xylazine 10 mg/kg) or fentanyl-medetomidine-midazolam mixtures (fentanyl 0.05 mg/kg, medetomidine 0.5 mg/kg, midazolam 5.0 mg/kg). Depth of anesthesia was assessed by monitoring pinch withdrawal and eyelid reflex. Opticorn-A ophthalmic ointment was applied to the eyes to prevent cornea dehydration during surgery.

A 4–5 mm-diameter craniotomy was made above the primary visual cortex in the left hemisphere under a dissection microscope by using stainless steel drilling bits. After opening the skull, the cortical surface was kept moist with Ringer’s buffer. Dura mater was carefully removed, and 2 µl SR101 (200 µM) was pipetted onto the brain surface and left for 5 min to stain astrocytes^81, 82^. The exposed cortex was sealed with a stack of 4 mm diameter glass coverslips glued together using Norland Optic Adhesive (Thorlabs, Newton, NJ, USA). In some experiments where an extracellular recording electrode was inserted into the craniotomy, part of the hole was left open and was covered only with low-melting point agarose. The rat was transferred into the imaging set-up, placed on a warming plate (37 °C), and head-fixed in a stereotaxic frame (David Kopf Instruments, Tujunga, CA, USA). Alternatively, a custom-made metal bar was attached to the skull with C&B-Metabond® adhesive cement (Parkell, Edgewood, NY, USA) to allow for subsequent head fixation during calcium imaging.

### *In vivo* imaging and electrophysiological recording

GCaMP2 expressing neurons in 100–300 µm depth (corresponding to layer 2) of the V1 primary visual cortex area were imaged using a two-photon microscope (Femto2D-Dual, Galvo-Resonant, Femtonics, Budapest, Hungary) equipped with a 16x water immersion objective excited by 875 nm laser line (Chameleon, Coherent, Santa Clara, CA, USA). GCaMP2 fluorescence was detected at 490–550 nm in either line-scan (32–156 Hz sampling rate) or full-frame (30–45 Hz sampling rate) mode. In experiments not performed on the day of the surgery, SR101 was reapplied intravenously to the tail vein in 10 mM (100 µl) at least 1 hour before the imaging^81^. SR101 has been proven not to label neurons^81^. Although oligodendrocyte labelling may occur after longer incubation^81^, oligodendrocytes are far less abundant^83^ in the superficial layers of the cortex compared to astrocytes. Therefore, it is safe to identify SR101 positive cells as astrocytes. SR101 positive astroglia were detected at 570–640 nm. The length of imaging recording sessions was 60 or 300 s, sessions were repeated 6–24 times during the anesthesia. In many cases, imaging sessions were made at different regions within the V1 area. Possible respiratory coupling with the fluorescent signal was controlled by parallel recording of breathing rate with a thermocouple (5TC-TT-J-36-36, Omega Engineering, CT, USA) inserted into the animals’ nostril. Local field potential (LFP) recordings were made in the same cortical layer with glass capillaries (3–6 MΩ) filled with Ringer’s buffer, inserted underneath the coverslip under visual guidance.

Drugs for perturbing astrocytic Ca^2+^ signalling were administered by two different methods. CBX was injected i.p. in 100 mg/kg concentration during SWA imaging. This experimental design allowed us to directly compare imaging and LFP data obtained in the absence and presence of the inhibitor. In contrast to CBX, the Cx43 antibody and thapsigargin do not cross the blodd-brain barrier, therefore these agents were applied directly onto the cortical surface after removal of the dura. Cx43 antibody (0.75 mg/ml, 10 μl) was applied 24 h before the experiment to allow diffusion into the tissue. The ability of the antibody to penetrate into the brain at this time scale was confirmed by applying an Alexa-488 labelled secondary antibody in the same concentration in a wild-type, age-matched rat. 24 h after the application Alexa-488 fluorescence was clearly detectable even at 800 μm depth. Thapsigargin (0.5 mM, 10 μl) was also applied onto the brain surface 30 min before the experiment. Under these conditions thapsigargin was demonstrated to specifically inhibit astrocytic Ca^2+^ signalling^44^.

### Data evaluation

Cells on full frame GCaMP2 fluorescence images were identified by semi-automatic Matlab scripts under manual supervision. The identified regions of interests (ROIs) contained the soma of the cells slightly expanded by 2–3 pixels in all directions to reduce the noise in fluorescence intensity. The identified ROIs (in full-frame scan mode) or the cells around the line scan segments (in line-scan mode) were visually validated and the cell type (neuron or astrocyte) was determined based on the absence or presence of SR101 labelling in the same ROI. Average intensity in the identified ROIs was then calculated followed by subtraction of and dividing by the average intensity of ROIs in the first 10 samples. Fluorescence peaks on the detrended ΔF/F0 traces were identified using 2*SD as amplitude threshold and 0.1 as slope-to-peak threshold. Detection thresholds were selected after manually revising calculated ΔF/F0 traces from 2 animals.

Spatial consistency of neuronal and astrocytic activities was assessed by calculating the number of manually validated cells showing recurrent Ca^2+^ transients in the 0.5–2 Hz frequency range during the 60–300 s recording sessions and dividing this value by the number of validated neurons or astrocytes within the same field of view.

The effect of the number of surrounding active cells on the activity of the enclosed neurons or astrocytes was calculated by determining the distance between all cell pairs in 5 different animals (N = 263478 cell pairs). Data from different experimental sessions on the same animals were pooled. Percentage of the enclosed cells’ involvement in the SWA was determined by counting the number of astrocytes or neurons showing SWA-related rhythmic activity and having 0, >1, >2, >3, >4 adjacent astrocytes or neurons within 100 µm. This count was divided by the number of all astrocytes or neurons having 0, >1, >2, >3, >4 adjacent astrocytes or neurons within 100 µm.

For spectral analysis LFP recordings were bandpass filtered using Bessel filters. Wavelet analysis was performed using complex Morlet wavelets.

Unless stated otherwise, data are expressed as means ± S.E.M. and were analyzed using one-way analysis of variances with Bonferroni post hoc tests (OriginPro 8.0). A value of P < 0.05 was considered significant.

## Electronic supplementary material

Supplementary information

Supplementary Movie 1

Supplementary Movie 2

Supplementary Movie 3
