# Supplementary material for: Extensive astrocyte synchronization advances neuronal coupling in slow wave activity in vivo
Source: Sci Rep. 2017 Jul 20;7:6018. doi: 10.1038/s41598-017-06073-7 (PMC5519671; doi:10.1038/s41598-017-06073-7)
Supplement: Supplementary file 1 — Supplementary information [file 41598_2017_6073_MOESM1_ESM.pdf]

Extensive astrocyte synchronization advances neuronal coupling in slow wave activity in vivo

Zsolt Szabó[1*], László Héja[1§*], Gergely Szalay[2], Orsolya Kékesi[1], András Füredi[3,6], Kornélia Szebényi[3,6], Árpád Dobolyi[4], Tamás I. Orbán[3], Orsolya Kolacsek[3], Tamás Tompa[2], Zsombor Miskolczy[5], László Biczók[5], Balázs Rózsa[2], Balázs Sarkadi[3], Julianna Kardos[1]

[*]These authors contributed equally to this work.

[1]Institute of Organic Chemistry, Research Centre for Natural Sciences, Hungarian Academy of Sciences, Magyar tudósok körútja 2, 1117 Budapest, Hungary

[2]Institute of Experimental Medicine, Hungarian Academy of Sciences, Szigony 43, 1083 Budapest, Hungary.

[3]Institute of Enzymology, Research Centre for Natural Sciences, Hungarian Academy of Sciences, Magyar tudósok körútja 2, 1117 Budapest, Hungary

[4]Laboratory of Neuromorphology, Department of Anatomy, Histology and Embryology, Semmelweis University, Tűzoltó 58, 1094 Budapest, Hungary

[5]Institute of Materials and Environmental Chemistry, Research Centre for Natural Sciences, Hungarian Academy of Sciences, Magyar tudósok körútja 2, 1117 Budapest, Hungary

[6]Institute of Cancer Research, Medical University Wien, Borschkegasse 8a, 1090, Wien, Austria

[§]To whom correspondence should be sent at the following address:

László Héja, Research Centre for Natural Sciences, Hungarian Academy of Sciences, Magyar tudósok körútja 2, 1117 Budapest, Hungary, email: heja.laszlo@ttk.mta.hu, phone: +36-1-382-6619

SUPPLEMENTARY INFORMATION

Supplementary figures

Supplementary Figure 1: GCaMP2 is not expressed in microglia and pyramidal cells. (A) Immunohistochemical staining of GCaMP2 protein with the astrocyte-specific GFAP, the reactive astrocyte-specific Iba1, the interneuron-specific GAD-67 and the neuron-specific NeuN markers in organotypic hippocampal slices. Scale bars: 50 µm (GFAP, Iba1 and GAD-67) or 200 µm (NeuN). **(B)** Expression of GCaMP2 protein in organotypic hippocampal slices. GCaMP2-expressing cells are not detected in the stratum pyramidale. G: dentate gyrus, R: str. radiatum, P: str. pyramidale, O: str. oriens. Scale bar: 200 µm.

Supplementary Figure 2: GCaMP2 is expressed both in astrocytes and neurons as demonstrated by simultaneous astrocyte-specific expression of the red RGECO protein. (A) Expression of CAG-GCaMP2 (green) and GFAP-RGECO (red). Neurons (some marked by arrows) express only GCaMP2, astrocytes (some marked by arrowheads) express GCaMP2 and RGECO. Scale bars: 50 µm. **(B)** 10-sec segments of $\Delta F/F_0$ fluorescent intensity traces of all identified astrocytes (n = 7) and neurons (n = 6) in the imaged area. All data were recorded from the V1 area of a P64 rat (n = 18 imaging session, 60 s each).

Supplementary Figure 3: Identification and classification of GCaMP2 expressing neurons and astrocytes on in vivo imaging data. (A) *Top:* Expression of GCaMP2 (green), labeling of astrocytes with SR101 (red). Both neurons (some marked by arrows) and astrocytes (some marked by arrowheads) express GCaMP2. *Bottom:* Zoomed-in image of the area marked by white rectangle at the top row. GCaMP2 expressing cells are classified based on the presence (astrocytes, A) or absence (neurons, N) of SR101 labelling in the same ROI. Scale bars: 100 µm.

Supplementary movies

Supplementary Movie 1: Fluorescent intensity changes of CAG-GCaMP2 in response to 1 mM ATP in organotypic hippocampal slices

Supplementary Movie 2: Fluorescent intensity changes of CAG-GCaMP2 in response to 100 μM Glu in organotypic hippocampal slices

Supplementary Movie 3: Fluorescent intensity changes of CAG-GCaMP2 in response to 10 μM ionomycin and 10 mM EGTA in organotypic hippocampal slices

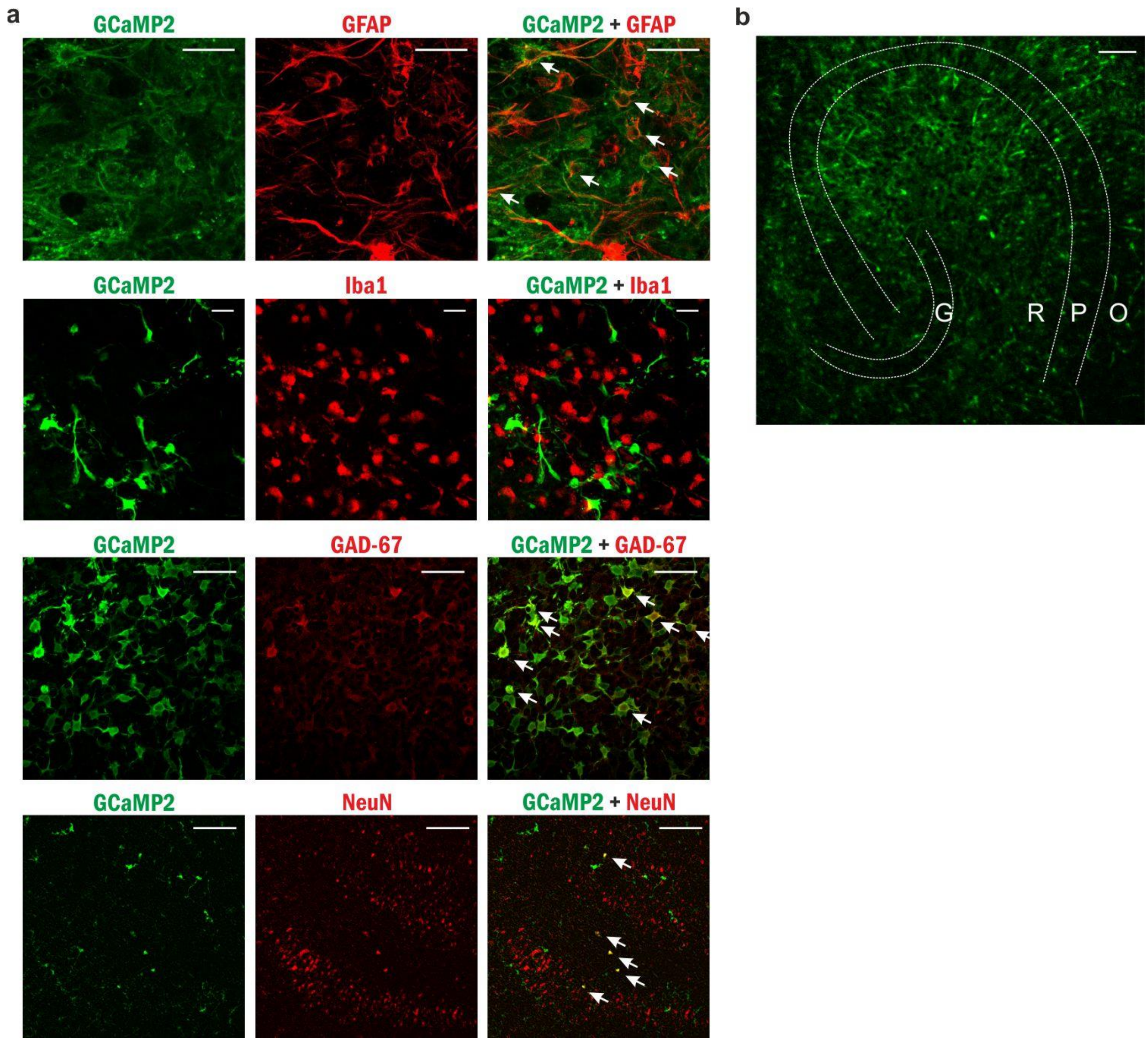

Supplementary Figure 1.

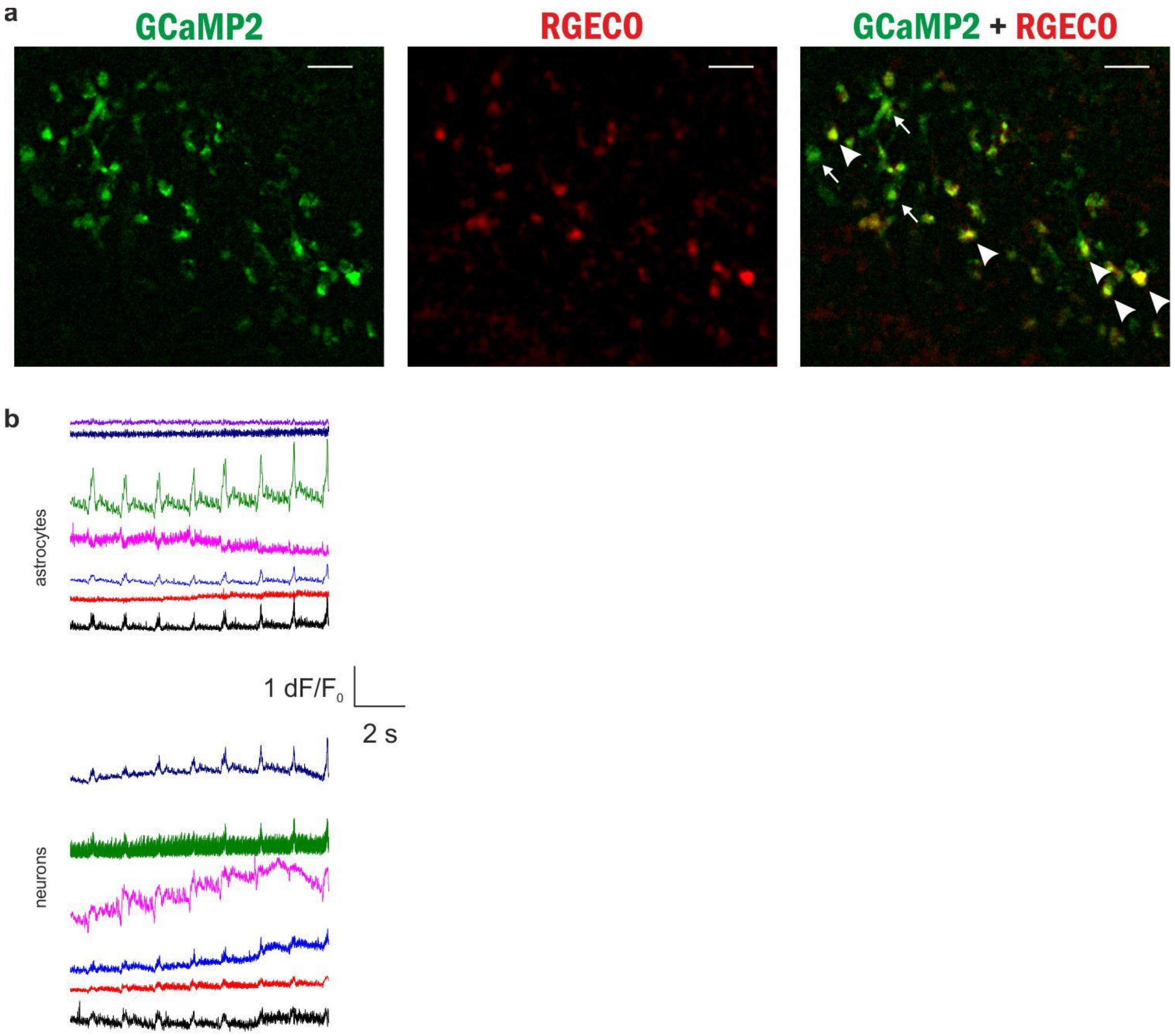

Supplementary Figure 2.

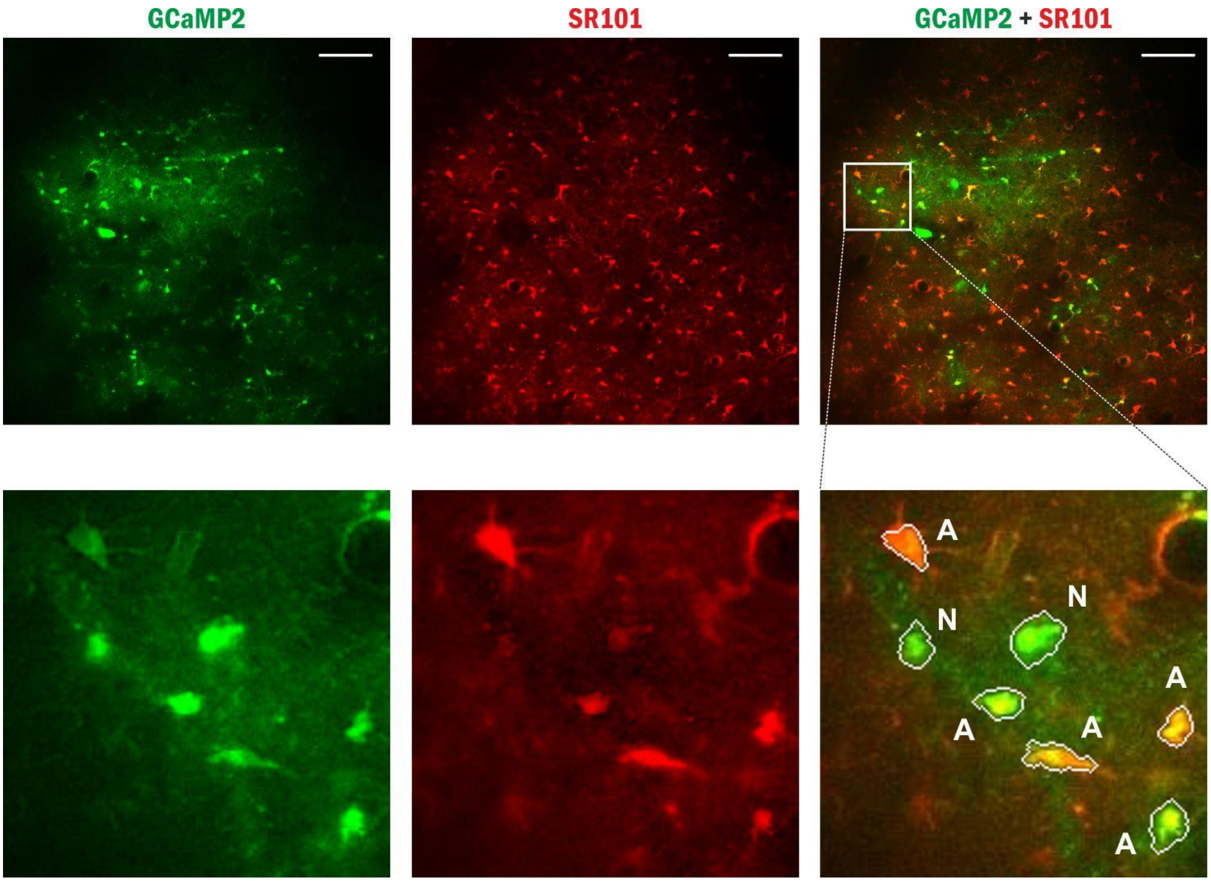

Supplementary Figure 3.
